# Cancer rehabilitation in clinical practice: a qualitative study exploring contact nurses’ views on prerequisites

**DOI:** 10.1186/s12912-025-02866-8

**Published:** 2025-02-27

**Authors:** Wenche Melander, Linn Rosell, Anna-Maria Larsson, Pernilla Lagergren, Marlene Malmström

**Affiliations:** 1 Department of Health Sciences, Lund University, Lund, Sweden; 2 Department of Surgery and Gastroenterology, Skåne University Hospital, Entrégatan 7, Lund, 222 42 Sweden; 3 Regional Cancer Center South, Lund, Sweden; 4 Division of Oncology, Department of Clinical Sciences Lund, Lund University, Lund, Sweden; 5 Department of Molecular Medicine and Surgery, Karolinska Institute, Stockholm, Sweden; 6 Department of Surgery and Cancer, Imperial College London, London, UK; 7 Institute for Palliative Care, Lund University and Region Skåne, Lund, Sweden

**Keywords:** Nursing, Cancer care, Rehabilitation, Needs, Qualitative

## Abstract

**Background:**

Individualized cancer rehabilitation should be an integral part of cancer care. Contact nurses play a key role in identifying patient needs and coordinating evidence-based interventions to support rehabilitation. However, cancer rehabilitation remains marginal in current practice, as contact nurses face challenges due to the lack of systematic processes for assessment, intervention, and follow-up, limiting its implementation across the cancer care trajectory. This study aims to explore contact nurses in cancer care views on their role in and prerequisites for cancer rehabilitation.

**Methods:**

Data were collected through 20 individual interviews with contact nurses working in Swedish cancer care and analyzed using qualitative content analysis.

**Results:**

A holistic approach to cancer rehabilitation was emphasized, yet establishing routines for assessment and addressing patients’ changing needs was described as challenging. Contact nurses experienced themselves as responsible for rehabilitation even though their role often was unclear. Rehabilitation was further seen as a process often determined by the medical trajectory rather than patients’ needs. To establish prerequisites for cancer rehabilitation supportive leadership and sufficient resources is essential.

**Conclusion:**

There is a gap between cancer rehabilitation guidelines and their implementation in clinical practice, emphasizing the need for structure to support contact nurses to provide evidence-based individualized cancer rehabilitation. To enable cancer rehabilitation, supportive leadership at the organizational level is essential for contact nurses to establish routines in their clinical practice. These routines should align the rehabilitation process with patient needs, ensuring that rehabilitative services are effectively integrated into regular healthcare visits.

**Clinical trial number:**

Not applicable.

**Supplementary Information:**

The online version contains supplementary material available at 10.1186/s12912-025-02866-8.

## Introduction

Currently there is a lack of knowledge and structures for how contact nurses in cancer care (CNCC) should systematically address and meet patients’ individual needs for cancer rehabilitation. Cancer rehabilitation aims to prevent functional impairments, and to ensure that the person maintains or regains the best possible functional capacity and quality of life, despite consequences of cancer and cancer treatment. The need for cancer rehabilitation varies based on different parameters such as the individuals’ personal conditions and preferences, social support [1], treatment, and type of cancer [2]. The CNCCs play a crucial role in cancer rehabilitation due to the continuous and trusting relationships they build with patients. Their responsibilities include responding to needs, coordinating, and following patients’ rehabilitation process [3]. However, previous research implies that assessment of needs is challenging in clinical practice both due to a lack of resources, e.g. the time needed to address patient needs, and that CNCCs are unable to refer patients to appropriate support [4].

## Background

The concept of CNCCs in Swedish cancer care has gradually been implemented over the last decade. The CNCCs are registered nurses (RNs) with a special assignment. In Sweden, RNs hold a bachelor’s degree in nursing with the possibility to undergo additional advanced level education for a specialist degree. In addition, Swedish universities offer specific CNCC courses, however none of these are mandatory for working as a CNCC. The CNCCs special assignment includes the responsibility to enhance individually tailored information and communication between the patient and the healthcare unit, enable accessibility, continuity and security, as well as strengthen patient participation. The assignment also outlines the necessity of evidence-based assessments, interventions, and following up on patient needs, such as rehabilitation [5]. According to a national mission statement developed in 2011, the CNCCs have a coordination responsibility throughout the clinical trajectory [6]. This entails care coordination, including referral to the appropriate healthcare professionals, as well as facilitating communication within the multidisciplinary team, which may also involve the patient [7]. However, the conditions for CNCCs to carry out their assignment vary from sporadic contact with the patient to holistic person-centered approach to care [8, 9]. This places high demands at a hospital organizational level to ensure that the CNCCs have sufficient competence [10] and optimally planned time and resources [3] for their assignment in cancer care. Many international role titles share similarities with the CNCC role in clinical patient care but are not equivalent, as they lack standardized definitions based on professional experience, educational degree, or national certification [11]. A recent scoping review highlights inconsistencies in advanced nursing roles in cancer care and underscores the need for international consensus on education requirements and regulatory standards for job titles [12]. The CNCCs often work at a surgical or medical/oncological department. However, the circumstances surrounding their assignment vary, which affects the prerequisites to carry out their task in accordance with the national description for CNCCs in cancer care [8, 9].

Between 74 and 98% of patients in Swedish cancer care are reported to have a CNCC, though the specifics of this role remain unclear [13]. Brynskog et al. (2024) note that while common aspects of the CNCC role, such as providing information and psychosocial support, are frequently practiced, other aspects, like cancer rehabilitation plans, are less commonly implemented. Most CNCCs report patient contact before, during, and up to three months after treatment, typically in person, by phone, or digitally. However, about one-third have no contact with patients 3–12 months post-treatment [9]. Persons diagnosed with cancer should according to the European Code of Cancer Practice be supported by the healthcare system throughout their cancer trajectory. The support includes an active approach to challenges such as functional and psychosocial concerns [14]. In cancer care patients’ needs are described to encompass care coordination, information, support of physical wellbeing as well as emotional support and self-care [15]. Assessment of rehabilitation needs ideally starts at the time of diagnosis and continues throughout treatment and follow-up [16]. Sweden’s national care program for cancer rehabilitation recommends the use of validated assessment tools to identify patients’ physical, psychological, social, and existential rehabilitation needs. The assessment tool is completed by the patient and serves as a basis for the conversation with the CNCC [17]. The structured assessment aims to capture needs as they arise, possibly reduce symptom burden, facilitate appropriate referrals to rehabilitation instances and improve patient outcomes [16]. However, previous studies describe unmet rehabilitation needs in the early phase of the cancer trajectory [18], but also during as well as after cancer treatment [2, 19]. In addition, patients perceive support from healthcare professionals as more apparent during active treatment [20] and diminishing over time, despite the ongoing need of support [20, 21]. Consequently, patients may experience a sense of being adrift or lost in their cancer rehabilitation [22].

Cancer rehabilitation is a multifaceted process, influenced by various perspectives, including those of the patient, healthcare organizations, and healthcare professionals. Still, it has been shown to play a marginal role in cancer care, thus increasing the risk of suboptimal rehabilitation outcomes [23]. In addition, the rising number of cancer diagnoses [24] and the focus on medical and treatment-driven approach to cancer care [23] highlight the importance of addressing the comprehensive needs of patients. This is further complicated by a lack of knowledge about cancer rehabilitation [25] and relevant guidelines [26] underlining the necessity for a stronger focus on the CNCCs’ perspective in the rehabilitation process. Consequently, enhancing the understanding of the nursing role and the prerequisites for cancer rehabilitation is crucial within clinical care.

### Aim

The aim of this study was to explore contact nurses in cancer care views on their role in and prerequisites for cancer rehabilitation.

### Design

This inductive interview study was conducted using an explorative design. Data were collected from individual interviews and analyzed using qualitative content analysis [27]. Reporting adheres to the Consolidated criteria for Reporting Qualitative research (COREQ) checklist [28].

### Context

Sweden has a healthcare system with 21 autonomous regions which manage the funding, organization and delivery of healthcare. Hospital care involves health and medical services requiring e.g., specialized medical equipment. Primary healthcare task is to prevent, assess, treat and rehabilitate patient needs that do not require hospital care. Due to a decentralized healthcare organization, delivery of service can vary across geographical areas [29]. In Sweden, National Guidelines for Cancer Rehabilitation [17] emphasizing the need for systematic processes to assess patient rehabilitation needs, initiate rehabilitation efforts and follow-up on rehabilitation plans, have existed since 2014. Cancer rehabilitation is divided into basic, special and advanced levels. Cancer rehabilitation is relevant across all levels of care, including primary, specialized, home, and palliative healthcare. Regional and municipal authorities must ensure patients’ rehabilitation needs are met. According to the national guidelines for cancer rehabilitation, the responsibility for patient’s basic rehabilitation needs is shared between the physician and a dedicated nurse, in Swedish cancer context referred to as a CNCC. Each diagnosis-specific care process should identify necessary specialized competencies at special and advanced levels. Some skills may be needed infrequently but across various cancer care processes, requiring clinics to share resources or access consultative support, potentially through specialized teams [17].

### Inclusion criteria

Registered nurses’ working as CNCCs across various diagnostic areas at Departments of Surgery or Oncology in county or university hospitals in southern Sweden were considered eligible for inclusion through a purposive sampling.

### Participants

The included registered nurses’ working as CNCCs, characteristics are described in Table 1.

**Table 1:** Interviewed CNCCs characteristics data (*n* = 20)

	Number of participants (total *n* = 20)
**Gender**	
Woman	20
Man	0
**Age (years)**	
38–47	5
48–57	8
58–66	7
**Hospital**	
University hospital	11
County hospital	9
**Department**	
Surgical	14
Medical/Oncological	6
Working as CNCC* (years)	
1–6	6
7–12	9
≥ 13	5
Working as CNCC* (% of work time)	
30–60	6
61–90	4
≥ 91	10
CNCC* education	
Yes	14
No	6

*CNCC = contact nurse in cancer care

Diagnostic groups: esophagus-, stomach-, liver-, pancreas-, biliary tract-, breast-, colorectal-, urology (prostate, bladder, penis, kidneys)-, lung-, head/neck- and gynecology cancers, malignant melanoma and sarcoma

**Fig. 1:**
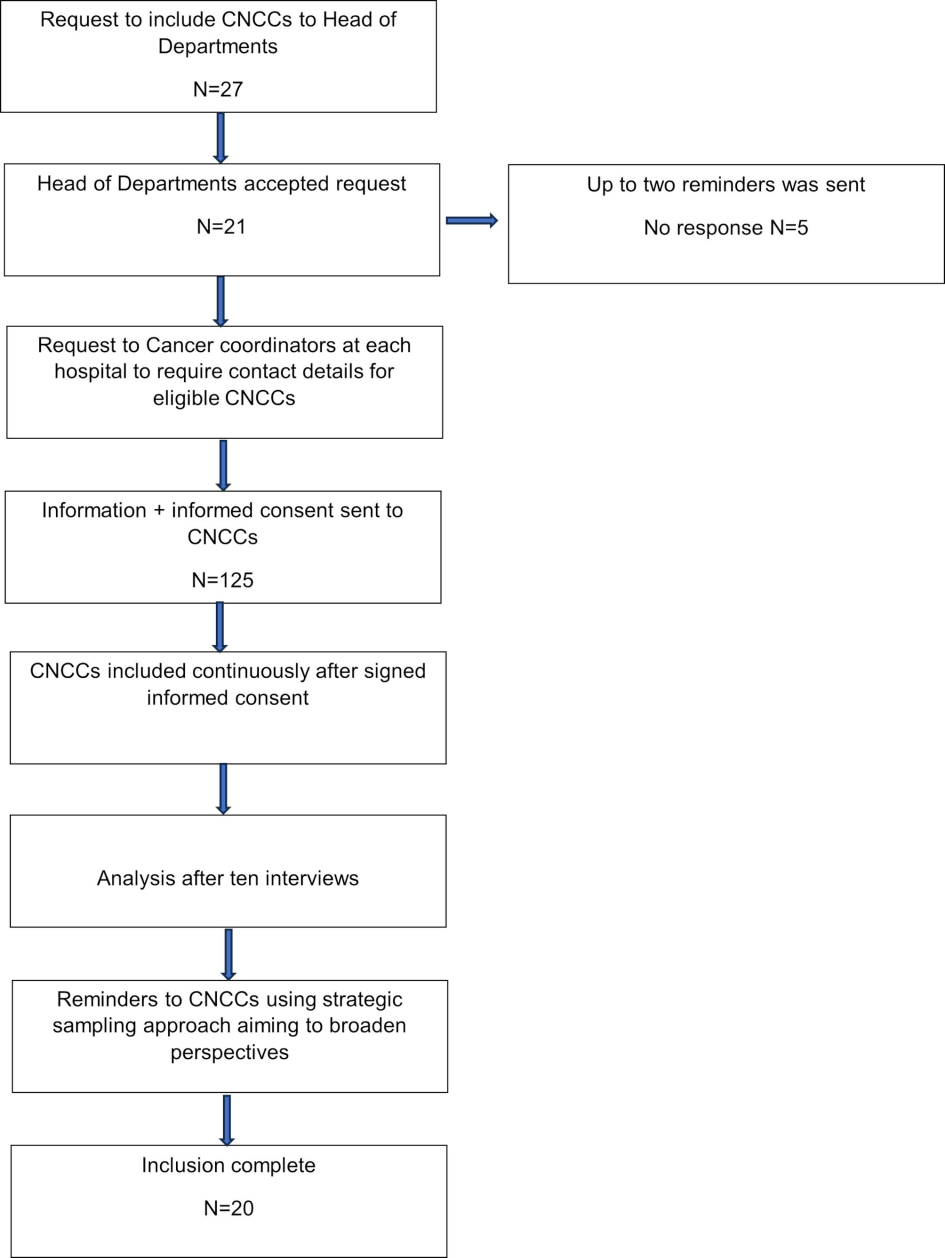
Flowchart inclusion procedure

Before start of inclusion, the head of departments gave permission to invite CNCCs to participate in the study. Thereafter cancer coordinators at each hospital provided contact information for eligible CNCCs. All CNCCs received information about the study by email together with an informed consent. The CNCCs were included consecutively and interviewed after they signed informed consent. To ensure the relevance of the interview guide, the first three interviews were conducted as pilot interviews. After the interviews, the guide was discussed in relation to the aim. The interview guide was deemed relevant for addressing the aim, and no changes were made. After half of the interviews were conducted, a preliminary analysis was performed. Subsequently, one reminder was sent using a strategic sampling approach to achieve variation and broaden perspectives. After 20 interviews were completed, no new perspectives emerged, and no further participants were included. Details on the inclusion procedure is described in Fig. 1.

### Data collection

A total of 20 interviews were conducted between March and June 2023 (19 digitally through video conference and one by phone). The interviews lasted between 39 and 70 min (median 57 min) and began with the researcher repeating the purpose of the study ensuring that the participant understood their rights as a participant in research. Demographic data were then collected (Table 1). A semi-structured interview guide based on literature in the research area, developed by two of the authors, was used (Additional file 1). The interviews started with an introductory open question “Can you tell me how/if you work with cancer rehabilitation in your work as a contact nurse in cancer care?” which was followed by probing questions such as “What support would you need to address challenges in relation to cancer rehabilitation?”. The researcher made a summary in the end of the interview, allowing the participant to add or correct information during the interview occasion, serving as a member check [28]. All interviews were conducted by one or two researchers (MM, LR, WM), all three of whom possess solid knowledge of the research areas and substantial experience and expertise in qualitative methodology. All interviews were audio recorded and transcribed verbatim, and printed interviews were validated against the recorded interviews.

### Data analysis

Data analysis was conducted using inductive qualitative content analysis [27]. The method was considered suitable based on the study’s purpose, the limited research on CNCCs’ views on cancer rehabilitation, and its ability to enable a deeper understanding of an unexplored phenomena. The analysis process was performed in phases. First the authors (MM, LR, WM) individually read and/or listened to the interviews several times to obtain a sense of the whole. Open coding was performed separately, and the codes were grouped into subcategories. Thereafter the authors (MM, LR, WM) discussed similarities and differences between subcategories and created a coding scheme which organized the content into categories. The abstraction process included moving back and forth between specific and general aspects. The relationship between the categories has been thoroughly discussed when developing main categories and subcategories and results were discussed among all co-authors until consensus was reached. The qualitative content analysis identified two main categories with two and three subcategories respectively (Table 2).

## Findings

**Table 2:** Overview of main categories and subcategories

1. CNCCs’ views on their role and responsibility in cancer rehabilitation	2. CNCCs’ views of organizational aspects influencing cancer rehabilitation
1:1 Diversity between perception and the scope of responsibility	2:1 Absence of overall organizational responsibility
1:2 Complexity in identifying and responding to patients’ individual needs	2:2 Value of leadership and resources
1:3 Importance of team collaboration	

### CNCCs’ views on their role and responsibility in cancer rehabilitation

A holistic approach to cancer rehabilitation was emphasized but establishing routines for assessment and addressing the patients’ changing needs was described as challenging. While the CNCCs viewed themselves as responsible for rehabilitation within their context, the relationship between their role, the broader team’s responsibilities, and the patient’s own role, was considered unclear.

#### Diversity between perception and the scope of responsibility

The CNCCs’ perceptions of cancer rehabilitation and their responsibility in this area were frequently discussed during the interviews. Cancer rehabilitation was described as a process encompassing assessment, interventions, and follow-up. When elaborating on the meaning of cancer rehabilitation, most CNCCs viewed rehabilitation as holistic, encompassing various aspects of recovery, with the primary goal to maintain health and wellbeing as well as to support return to daily life. While some CNCCs described rehabilitation needs as generic and primary linked to diagnosis and treatment, others emphasized that rehabilitation needs were individual. Further, several CNCCs elaborated on the distinction between nursing and rehabilitation, with the majority describing that this distinction was both unclear and challenging. Some argued that the two perspectives were so closely intertwined that separating them was not only difficult but also irrelevant.

“*Then*,* in a sense*,* that is what we do*,* the nurse in her conversations with the patient*,* it is rehabilitation. That’s what we’re talking about*,* how are you? How will you proceed? So maybe it’s just that the word rehabilitation is not in my head*,* but all I really do when I talk to a patient is rehabilitation*”. (P:16)

The CNCCs often described their profession as one involving the most frequent contact with patients throughout the cancer trajectory, positioning them in a coordinating role starting already at the time of diagnosis. However, perspectives varied regarding when their responsibility ended. The CNCCs elaborated on their responsibility for rehabilitation throughout the cancer trajectory, describing it as undefined but providing examples of their actions. These included informing patients about rehabilitation, motivating them, offering rehabilitative interventions, and helping the patient navigate the healthcare system.

#### Complexity in identifying and responding to patients’ individual needs

While most CNCCs recognized the various elements included in cancer rehabilitation, the majority described a lack of routines related to rehabilitation or challenges in maintaining existing ones. Practices for using assessment tools varied greatly and some CNCCs emphasized the advantages of using assessment tools to identify rehabilitation needs. These tools provided a structured approach for addressing the patient’s most pressing problems and facilitating timely support. However, establishing consistent routines for rehabilitation was described as challenging, despite the awareness of the importance of repeated assessments identifying patient’s changing needs. After initial assessment, some CNCCs reported occasional follow-up with the patient during the cancer trajectory. However, this latter part of the rehabilitation process generally lacked clear clinical routines.

Some CNCCs stated that their response to patients’ needs was influenced by their work experience and their so called “clinical eye”, which often centered on common needs associated with specific diagnoses. Their knowledge was also drawn from various sources, such as lectures, journal clubs, collaboration with rehabilitation professionals, advice from experienced colleagues and national care guidelines. Despite this, they viewed competence development as an ongoing process and emphasized the importance of staying up to date, even though this often could be challenging. One CNCC said,

*“Sure*,* but partly I still feel like we’re trying to work evidence-based*,* but it’s hard to know. In some aspects*,* maybe we just stick more to our usual routines”* (P:4).

Most CNCCs stated that it was easier to identify diagnosis-related common rehabilitation needs that they could manage themselves, such as providing information on rehabilitation and self-care, promoting physical activity and addressing pain management. In contrast, they highlighted the challenges of addressing more complex or sensitive needs. As one CNCC expressed *“… well*,* I think that sex is incredibly difficult to talk about [laughs]*,* I get very uncomfortable”* (P:13). The CNCCs’ described a sense of uncertainty when dealing with sensitive needs, referred to as complex needs, and a lack of definition of what “complex needs” meant. This ambiguity made it difficult to identify patients with the most profound needs, often resulting in ad hoc referrals to rehabilitation professionals or specialized cancer rehabilitation services.

#### Importance of team collaboration

Collaboration was described by the CNCCs in three aspects, with the patient-responsible physician, the team and the patient. The team constellation was described differently depending on the organizational unit, i.e. including various rehabilitation professionals, other registered nurses, and medical secretaries. This means that the CNCCs views of the availability of rehabilitation resources and the preconditions for rehabilitation differed.

Challenges related to team collaboration included a noticeable lack of clarity regarding the division of responsibilities, as described by one CNCC

“Is *it us nurses or is it the doctors or is it a joint responsibility? No*,* I can’t really answer that. Or the patient himself [laughs]. No*,* I think it’s more likely that we*,* nurses are actually responsible*,* but…”* (P:4).

A close alliance and shared responsibility between the CNCC and physician, along with having an assigned patient-responsible physician, were described as a prerequisite for successful teamwork. However, the CNCCs elaborated upon different views of the prioritization of rehabilitation.

“*Well*,* from the doctors’ point of view*,* I don’t think they are thinking about cancer rehabilitation. I don’t think so. I haven´t experienced it*,* but on the other hand*,* they are very concerned about the patient’s well-being”* (P:5).

Collaboration with the team was considered essential to create common routines, fostering knowledge development, building trust, and creating an open climate for discussion. Regular weekly or monthly meetings with the whole team were highlighted as good examples, enabling the provision of additional rehabilitation for patients.

The patient was considered a partner in the team, having a crucial role in accessing information, filling out assessment forms, and being motivated to conduct their rehabilitation.

It was described that most patients were presumed to take responsibility, but at the same time the CNCCs elaborated on the difficulties for some patients to take this responsibility, e.g. in the case of depression. These patients were expected to have an extended need for support.

### CNCCs’ views of organizational aspects influencing cancer rehabilitation

The cancer trajectory was described as an overarching concept encompassing the entirety of a patient’s cancer care journey. Within this trajectory, rehabilitation emerged as one of several interconnected processes influenced by parallel processes such as the medical treatment plan and the patient’s personal experience. Sufficient, leadership and adequate resources were highlighted as essential for CNCCs to establish the necessary structure and conditions to support rehabilitation effectively.

#### Absence of overall organizational responsibility

The CNCCs described a lack of a comprehensive approach to rehabilitation, noting that it was primarily centered around the medical process. Patients’ needs were often assessed in conjunction with routine healthcare appointments. While CNCCs mentioned monitoring the patient´s condition during these visits, opportunities to evaluate or conduct rehabilitation interventions were limited, as the appointments typically focused on delivering diagnoses or conducting post treatment follow- up. Patients were in general described as being most engaged at the beginning of the cancer trajectory. Moreover, their well-being during treatment was noted as a significant factor influencing their capacity to participate in rehabilitation efforts. One CNCC stated

*“There aren’t many who want to join group rehabilitation*,* or… perhaps they’re undergoing radiation treatment*,* and if they’re undergoing radiation*,* they don’t want to go and exercise. You can’t do two things*,* so they should focus on the radiation. That’s what I think*,* anyway”* (P:16).

The CNCCs described their role as being pivotal in holding together the fragmented cancer trajectory, largely due to their ability to provide continuity. This continuity was emphasized as essential for identifying and following up on rehabilitation needs, offering patients a sense of security. Moreover, it was noted that continuity influenced the scope of rehabilitation interventions that could be delivered. However, some CNCCs highlighted barriers to achieving this, such as work schedules, which were aligned with physicians’ availability, creating an obstacle for CNCCs to meet with patients.

As the cancer trajectory progressed, the fragmentation of care became more evident, with an increasing shift of responsibility from specialist care to the patients. For example, medical follow-up visits became less frequent, leaving the patient responsible to contact the CNCC if their rehabilitation needs changed, or new needs arose. In contrast, the CNCCs noted that a deeper understanding of patients’ needs could enable more individualized and tailored rehabilitation. However, opinions varied regarding which patient characteristics influenced rehabilitation. Some stated that patients’ needs were closely linked to factors such as age, while others emphasized personal factors, such as the ability to articulate needs.

*“Sexologist*,* I have never received a question from patients… because I think we simply have too old patients…”* (P:5).

The fragmentation of care was also described from the nursing perspective, as CNCCs noted that they were often only responsible for only specific parts of the cancer trajectory. While patient rehabilitation was generally managed by the treating unit, the responsibility became uncertain when patients changed care providers. This uncertainty made it challenging to ensure a comprehensive rehabilitation process. The CNCCs emphasized the need for improved collaboration, including clear referral procedures across organizational boundaries, for example referrals to a specialized cancer rehabilitation unit or a primary health provider. However, they noted that collaboration became increasingly difficult as the organizational distance between units grew.

“*You would almost need a meeting with them [Primary Health Care]*,* a bit of what they are capable of as well. For that… What they can do and can take over and so on. So that you see the possibilities*,* because maybe that’s something we miss too. And then it’s difficult*,* because we have patients all over the Region. So that there are many primary health care centers involved*” (P:2).

#### Value of leadership and resources

Leadership and adequate resources were identified as essential for enabling CNCCs to effectively work with cancer rehabilitation. Despite this, CNCCs consistently described a lack of these critical factors within the organization.

Their perception of organizational responsibility for rehabilitation varied greatly. Many CNCCs stated that hospital management held the primary responsibility for ensuring prerequisites for cancer rehabilitation. However, limited resources posed substantial challenges to their ability to provide rehabilitation. Key identified resources included sufficient staffing, dedicated time, structured patient meetings, and appropriate physical space to enable one-on-one conversations with patients. A particularly crucial factor was the number of patients assigned to each CNCC, which varied greatly and significantly impacted their capacity to deliver rehabilitation.

“*We are supposed to do a lot. Especially when you’re supposed to meet a person individually*,* then you need time. You need… at the same time something is beeping here and there and then the phone rings. Then you need peace and quiet*,* and time. Maybe we can’t work the way we work now…*” (P:15).

The work with cancer rehabilitation was described as a responsibility assigned to the CNCC without the organization ensuring relevant prerequisites. Several CNCCs expressed that cancer rehabilitation was often deprioritized within the organization due to resource constraints and a predominant focus on the biomedical perspective in the cancer trajectory.

*“We have to take care of our patients*,* we have to do well in our lists*,* we have to be available*,* we have to work off our queues for diagnostic investigations*,* it’s not so clear for this rehabilitation… Rehabilitation*,* it’s not as clear*,* we don’t have a queue so*,* in that way*,* you don’t see it in the same way*,* if you know what I mean?”* (P:11).

Leadership was highlighted as a requirement for CNCCs’ work with rehabilitation. It was stated that, at the management level, knowledge of cancer rehabilitation and its benefits, as well as understanding the CNCC’s responsibilities and role herein, was essential for prioritization of rehabilitation. Several CNCCs described positive examples of supportive management, including responsiveness, education initiatives, and encouragement of collaboration between units. However, many CNCCs also emphasized a lack of managerial support, which often resulted in rehabilitation not being prioritized or formally required.

Organizational support was identified as essential for fostering collaboration between professions and units both within and outside the hospital, to introduce initiatives that promoted rehabilitation. Challenges in implementing new routines were often linked to time constraints, logistical complexities, and the additional strain of introducing multiple new work methods simultaneously. To enable sustainable change, leadership that provided support, competence development and knowledge about implementation was highlighted. Implementation of already existing routines from e.g. research projects or healthcare development projects was claimed to be a success factor. This was suggested to be done by the organization by bringing in persons with knowledge, such as specialist training, who could drive the change of routines.

## Discussion

Contact nurses in cancer care have a significant role in assessing patient needs and ensuring evidence-based interventions to promote cancer rehabilitation throughout the cancer trajectory. Despite this, there is currently a lack of knowledge and structures to effectively ensure effective rehabilitation assessment, interventions, and follow-up. This exploratory qualitative study provides in-depth knowledge into CNCCs’ views on their role and prerequisites for cancer rehabilitation. It highlights both the individual responsibilities of CNCCs and the organizational factors influencing rehabilitation, revealing a lack of a comprehensive approach due to unclear responsibilities, priorities, routines, and leadership in the patients’ fragmented cancer trajectory.

The CNCCs in the present study often described cancer rehabilitation from a holistic perspective including assessment, intervention and follow-up of patients’ needs, which closely aligns with the nursing process [30]. This could explain our finding describing that the distinction between nursing and rehabilitation was often seen as unclear, and some CNCCs stressed that although this was perceived as challenging, it was irrelevant to separate the perspectives since both address patient needs. Nurses play a crucial role in improving healthcare delivery by ensuring continuity, advocating for patients [31], often focusing on assessing and managing the patient’s symptoms and needs [10]. However, patients desire personalized care that is tailored to their individual situation and needs [32]. This includes care coordination, information, support for physical and emotional wellbeing, self-care, and treatment, including symptom management [15]. Yet, meeting each patient’s needs requires effective routines for cancer rehabilitation. Despite the CNCCs stating the use of assessment tools systematically facilitated evaluation of the patient’s needs, it was evident there was often a lack of routines related to structured assessment. At the same time, the CNCCs reported that they often relied on their so called “clinical eye”, based on their work experience which related to a specific diagnosis. However, this was also described to increase the risk of overlooking complex or sensitive needs. Mortensen and colleagues [33] described that nurses commonly assessed patient needs related to a specific diagnostic group, which is often linked to physical and functional requirements. The CNCCs in our study identified age as a factor influencing patients’ needs, potentially leaving some groups underserved. This may impact physical and cognitive health, functional status, and overall quality of life [34]. Mortensen and colleagues [33] also revealed that the nurses themselves experience conversations about sensitive topics as difficult, which was also reported in our study where the CNCCs elaborated upon the challenge to address complex or more sensitive needs. This study also elaborated on nurses claiming that discussions related to sensitive topics could cause the patient more harm than good. This could be interpreted as nurses assessing the needs they are familiar with, staying within their ‘professional comfort zone’, which highlights the need for educational initiatives within the field of conversation methodology.

The team providing cancer rehabilitation often consists of various health care professions. The collaboration within the multidisciplinary team enables a comprehensive approach to the patients’ individual needs requiring a shared understanding of roles and responsibilities [35]. The CNCCs in our study viewed themselves as responsible for rehabilitation but reported a lack of clarity regarding the division of responsibilities within the team. This is supported by Olsson Möller and colleagues [23], noting that nurses were claimed responsible for identifying needs, but the overall responsibility in the rehabilitation process remained unclear. Kerr and colleagues [31] describe the nurses in cancer care as the glue that holds the team together which could result in a growing dependence on nurses, potentially leading to other team members not fulfilling their role. Our study highlighted open discussions about, for example routines, as a factor facilitating team collaboration. Nevertheless, previous research highlights that unclear collaboration and responsibility within the team, as well as between departments in the organization, create a barrier for effective rehabilitation throughout the patient’s cancer trajectory [23].

Cancer rehabilitation should be integrated throughout the cancer trajectory to support patients [16]. Despite this, our study found that the rehabilitation process is influenced more by the healthcare system’s structure, knowledge, priorities, and resources than by patient needs. This is exemplified by Brynskog and colleagues [9] describing approximately one third of CNCCs having no contact with patients in cancer care 3–12 months post treatment. Furthermore, research findings indicate that the biomedical perspective often dominates the cancer trajectory [23, 25]. Patients consistently report unmet needs of rehabilitation throughout their cancer trajectory [2, 18, 19]. Despite this, Mortensen and colleagues [33] showed that nurses often hesitate to assess certain needs, such as emotional needs after surgery. Witte and Handberg [25] also found that nurses avoid assessing needs early in the trajectory as they meant that the patients are in shock, despite early need assessment through prehabilitation having been shown to optimize outcomes and provide timely referrals [16]. Hence, it is time to take action in person-centered care, prioritizing patient needs as the driving force rather than organizational structures, conditions, or professional assumptions about the appropriate timing for assessing needs.

Our study shows that leadership with knowledge about rehabilitation, sufficient resources, and structure for implementation of routines was emphasized as essential for CNCCs’ engagement in rehabilitation. Support from leadership facilitated collaboration between professions, but also between units, both within and outside hospitals. Justesen and colleagues [3] also highlight that effective leadership, along with continuity, education, time, and resources, is essential for optimizing the use of nurses’ professional skills in cancer rehabilitation. Therefore, it is fundamental to ensure knowledge-driven leadership that enables CNCCs to have well-functioning routines and structure for individually tailored cancer rehabilitation.

The national care guidelines for cancer rehabilitation in Sweden outline interventions and the roles of healthcare professionals throughout the care trajectory, presenting a complex multidisciplinary approach [17]. This complexity can impact nurses’ rehabilitation efforts, as nurses in cancer care find it challenging to implement such guidelines in clinical care [26]. Effective implementation relies on supportive leadership and organization [36], as well as time and personnel resources, with simpler guidelines being more likely to succeed [37]. To successfully implement guidelines, these needs to be adapted to the clinical context and the organization must ensure active engagement of persons involved [38]. To implement routines or practices based on guidelines, it is necessary to address all levels of an organization, from the individual level—represented in our study by the nurse—to the organizational leadership level. At all levels, potential barriers and facilitators need to be identified before implementation [38]. Rehabilitative interventions based on the patient’s needs are outlined in the Swedish National mission statement [6], and the CNCC is identified as a key person with a written assignment that includes clinical work with cancer rehabilitation [5]. However, the results from our study indicate that the nurse’s role, as well as routines based on individual patient needs, teamwork, and the prioritization of cancer rehabilitation within the cancer trajectory are only partially implemented.

### Strengths and limitations

A methodological strength of this study is that CNCCs who worked in six different hospitals were included, within both surgical and medical/oncological care, and encompassing a total of 16 different diagnostic groups, which allowed for a variation of perspectives. However, we did not analyze the data specifically based on demographic variables, as our focus was on capturing a broad range of experiences rather than demographic-specific differences. Five heads of departments did not respond to the request for inclusion, and one declined. Additionally, all participants in this study were female. This could be considered a limitation, as the lack of responses and the exclusion of certain perspectives, including male viewpoints, may have influenced the findings. To enhance trustworthiness, a purposive sampling strategy was used as we aimed to include participants with the most expertise in the research area. To ensure credibility, the authors had continuous discussions during the interviews to confirm that the data addressed the research purpose [39]. Participants were recruited through email, potentially skewing the results as CNCCs with stronger feelings about their experiences might have been more inclined to participate. On the other hand, the participation of a substantial number of CNCCs (*n* = 20) yielded rich interview data. This suggests that the findings could be transferable to a Swedish context, as they illuminate CNCCs’ views regarding their role in and prerequisites for cancer rehabilitation.

The interviews were conducted digitally, potentially enabling CNCCs who might not have been able to participate due to time constraints or geographical distance to take part in the study [40]. In some interviews, two interviewers participated, with one conducting the interview and the other observing. Having two interviewers can enable a power imbalance in favor of the interviewers [41]. Therefore, we addressed this by informing the participant at the beginning of the interview about the roles of each person and asking for the participant’s consent. Since two of the authors (LR, WM) work in cancer care, there were participants with whom they could potentially have a pre-existing relationship. To avoid bias or assumptions, and to ensure credibility, these participants were interviewed by another researcher (MM) which could be considered a strength. Another strength is that, to ensure accurate understanding, the interviewer provided a summary at the end of each interview for participants to amend, which, based on COREQ criteria, serves as a form of member-checking [28]. Three authors (MM, LR, WM) conducted the analysis to enhance comprehensibility and offer robust data interpretation [39]. Reliability was strengthened through joint, transparent discussions among all authors which resulted in collaborative agreements on essential main categories and sub-categories [27]. The results are strengthened by representative quotations, enhancing the conformability [39].

## Conclusion

According to the national guidelines for cancer rehabilitation, CNCCs have an essential role, being responsible for assessment, intervention, and follow-up of patients’ rehabilitation needs. However, our study demonstrates a gap between national guidelines for cancer rehabilitation and their implementation in clinical practice, emphasizing the need for structure and routines to enable CNCCs to provide evidence-based individualized cancer rehabilitation. To facilitate a rehabilitation process tailored to the patient’s individual needs, healthcare routines must be established to prioritize needs-driven care over organizational structures. Structured assessment tools facilitate need evaluation and should be used continuously throughout the cancer trajectory. Structured assessment tools ensure that all patient needs are identified, including complex needs which are often described as challenging to address. To ensure the CNCCs develop well-functioning routines and structures for individually tailored cancer rehabilitation, supportive leadership and a healthcare organization that prioritizes and demands rehabilitation in clinical cancer care are fundamental.

### Implications

This study provides valuable insights into CNCCs’ perspectives on cancer rehabilitation. Further research is needed to explore organizational views and the perspectives of other healthcare professionals, including those in primary care, to gain a holistic understanding of the rehabilitation trajectory. The findings also highlight the central role of nurses and the necessity of organizational support for structured, patient-centered care. The clinical implications underscore the importance of integrating cancer rehabilitation into clinical practice, requiring organizations to recognize CNCCs’ roles and prioritize rehabilitation within healthcare settings.

## Electronic supplementary material

Below is the link to the electronic supplementary material.

**Supplementary Material 1**: **Additional file 1**: Interview guide.

## Data Availability

The datasets used and/or analysed during the current study are available from the corresponding author on reasonable request.

## Abbreviations

CNCC: Contact nurse in cancer care
COREQ: Consolidated criteria for reporting qualitative research
RN: Registered nurse
