# Supplementary material for: Cancer rehabilitation in clinical practice: a qualitative study exploring contact nurses’ views on prerequisites
Source: BMC Nurs. 2025 Feb 27;24:223. doi: 10.1186/s12912-025-02866-8 (PMC11866813; doi:10.1186/s12912-025-02866-8)
Supplement: Supplementary file 1 — Supplementary Material 1: Additional file 1: Interview guide. [file 12912_2025_2866_MOESM1_ESM.docx]

Additional file 1 Interview guide

Interview guide

Open introductory question

Can you tell me how/if you work with cancer rehabilitation in your work as a contact nurse?

Suggestions for questions based on the respondent's answers:

Responsibility - Tell us who you think is responsible for cancer rehabilitation?

Assessment - Tell us how you assess the patient's rehabilitation needs (Assessment instruments? Structure? Evidence-based measures? Decision support linked to assessment? Repeated assessments/follow-up? Documentation/rehab plan/My care plan)

What is included in cancer rehabilitation? (living habits, physical activity, psychosocial support)?

Where does the cancer rehabilitation take place? Primary care/specialist care? (Referral sent? Active handover?)

Who should have access to cancer rehabilitation (-curative vs. palliative patient, within county, outside county?)

The rehabilitation process, when does rehabilitation start and when does it end? (what rehabilitation do you do at different times, does it differ?)

Prerequisities for cancer rehabilitation?

How do you feel the conditions for rehabilitation are?

Describe the support from the organization in terms of cancer rehabilitation?

What support would you need to deal with challenges/implement a change?

Do you and your colleagues have the necessary skills/competence?

Who is responsible for cancer rehabilitation in your organization?

How is cancer rehabilitation prioritized/valued in your organization?

Visionary question

There is a model for cancer rehabilitation that is being tested at some units, providing structured individually adapted evidence-based cancer rehabilitation throughout the cancer trajectory.

-What do you think would be needed in your organization for such a model to work?
